# A Comprehensive Examination of Age-Related Lower Limb Muscle Function Asymmetries across a Variety of Muscle Action Types

**DOI:** 10.3390/geriatrics9030079

**Published:** 2024-06-09

**Authors:** Kylee L. Heap-Eldridge, Brennan J. Thompson, Cody Fisher, Talin J. Louder, Jon Carey

**Affiliations:** 1 Kinesiology and Health Science Department, Utah State University, Logan, UT 84322, USA; 2 Movement Research Clinic, Sorenson Legacy Foundation Center for Clinical Excellence, Utah State University, Logan, UT 84322, USA

**Keywords:** muscle imbalance, aging, older adults, strength, power, velocity

## Abstract

Previous research has found that lower limb muscle asymmetries increase with age and are linked to fall and injury risks. However, past studies lack a wide variety of muscle function modes and measures as well as comparison to a comparable younger age group. The purpose of this study was to examine age-related lower limb muscle function asymmetries across a variety of muscle action types and velocities in young and old adults. Lower limb balance, strength, power, and velocity were evaluated with concentric, isometric, isotonic, and eccentric muscle actions during a single-leg stance test and on single- and multi-joint dynamometers in 29 young (age = 21.45 ± 3.02) and 23 old (age = 77.00 ± 4.60) recreationally active men and women. Most (15 of 17) variables showed no statistical (*p* > 0.05) or functional (10% threshold) limb asymmetry for either age group. There was a significant main effect (*p* = 0.046; collapsed across groups) found for asymmetry (dominant > non-dominant) for the isotonic peak velocity variable. There was a significant (*p* = 0.010) group × limb interaction for single-joint concentric peak power produced at a slow (60 deg/s) velocity due to the non-dominant limb of the young group being 12.2% greater than the dominant limb (*p* < 0.001), whereas the old group was not asymmetrical (*p* = 0.965). The findings of this investigation indicate there is largely no age-related asymmetry of the lower limbs across a range of muscle function-related variables and modes, with a couple of notable exceptions. Also, the significant asymmetries for the isotonic peak velocity variable perhaps show the sensitivity of this uncommonly used measure in detecting minimally present muscle function imbalances.

## 1. Introduction

Aging is associated with a substantial deterioration of neuromuscular function. Muscle power, the ability to generate force rapidly [1], and muscle strength, the maximal amount of force produced from a muscle either concentrically, eccentrically, or isometrically [2], all significantly deteriorate with advancing age [2,3,4]. Given that these muscle function parameters are positively associated with balance, ambulation, and locomotion in older adults [2,4,5,6], impairments would naturally lead to physical dysfunction in daily living activities, including slowed gait and less postural stability during dynamic tasks, resulting in a greater risk for falls. These effects have a profound negative impact as older adults that experience falls often experience accelerated deteriorations in health, quality of life, and survival rates [5,7]. In fact, in the United States, approximately 10–25% of falls in individuals aged ≥ 65 were due to gait problems and muscle weakness [8].

Thus, the preservation or even augmentation of force- and velocity-based capacities in older adults is of paramount importance as it pertains directly and significantly to their functional capacity, independent living ability, and quality of life. Although all force-related parameters are important for their contribution to functional tasks, it has been observed that the rapid force characteristics, which include power as well as a variable known as rate of torque or force development (RTD or RFD), may be more functionally relevant than maximal force capabilities alone due to their inherent time-dependent attributes [9,10,11,12]. In addition to force-based measures, previous studies have found that contraction velocity also significantly declines with age [13,14]. However, dynamometer-based muscle function studies have largely overlooked velocity-specific variables (e.g., peak velocity) as they may be less convenient to obtain from dynamometers than traditional measures such as torque or power. Although a number of studies have reported large age-related declines in muscle force, velocity, power, and rapid force characteristics [3,11,12,13,15,16], relatively few studies have investigated the inter-limb asymmetries of these characteristics in an aging context. For example, to our knowledge, no study has examined the effects of aging on maximum contraction velocity with regards to limb dominance. Muscle asymmetries are defined as the percentage difference in the muscle performance parameter (e.g., strength, power, etc.) between the dominant and non-dominant limbs [17]. For the purposes of this study, functional asymmetries are defined as greater than or equal to 10% inter-limb difference [1,2,18]. In addition to the magnitude of force-based measures, the force-related muscle asymmetry between limbs may be an important measure to consider. For example, in comparison to older adults who had not experienced an unexplained fall in the past year (13%), 60% of those who had sustained falls had asymmetries (>10% difference) in lower limb muscle power [1,2]. While general muscle power can be used to predict the fall risk of older individuals, muscle power, paired with lower limb asymmetry, may be more predictive of future falls than more traditional measures [5,17]. Moreover, in comparison to older adults who had not experienced two unexplained falls in the past 6 months, those who had experienced two or more falls had greater strength bilateral asymmetry (>10% difference) in the quadriceps and hamstrings [5]. Thus, combining muscle function measures along with limb asymmetries may be a complimentary means to increase sensitivity of identifying muscle function-related impairments in older adults who may be at more risk for physical dysfunction.

Previous studies have mostly examined age-related lower limb muscle asymmetries during concentric, eccentric, or isometric contraction types at a single velocity [5,6,18,19]. Prior studies [5,18,20] have also involved either single- or multi-joint testing, but have not examined comparisons of both. Moreover, other studies [5,21,22,23] involved either young or older adult groups, but have generally lacked a comparison of the two age groups. The singularity of the earlier studies regarding aging, asymmetries, and joint comparison limits the ability to determine comprehensive age-related effects on muscle asymmetries. Also, studies and meta-analyses [3,15] have commonly examined the age-related declines in muscle force or rapid force characteristics without regard to limb dominance, which would be an important factor to consider in understanding the impact of age-related limb asymmetries and its characteristics pertaining to function.

Thus, the current literature is limited regarding the examination of limb asymmetries and comparing them in younger and older adults using a comprehensive lower limb assessment battery that investigates multiple types of muscle actions (concentric, eccentric, isometric, and isotonic; single-joint and multi-joint), along with different velocities. Such an investigation would be important because it would help determine the extent to which limb asymmetries are present across a range of muscle action types and measures, which would help to elucidate what measures may be more sensitive to age-related limb asymmetry consequences and changes. This information could help clinicians and researchers provide more targeted interventions to better address inter-limb imbalances as a means to lead to improved muscle function, as well as reduced disabilities and fall risk in older adults.

Therefore, the purpose of this study was to examine age-related lower limb muscle function asymmetries across a variety of muscle action types, measures, and velocities in younger and older adults. Lower limb muscle strength, power, and velocity were evaluated with concentric, isometric, isotonic, and eccentric muscle actions on single- and multi-joint dynamometers. Lower limb balance function was also evaluated using a single-leg stance test (SLT). We hypothesized that the lower limb asymmetries would be greater for older adults, and that these imbalances would be more pronounced for rapid force variables (power, RTD), for multi-joint vs. single-joint tasks, and for faster vs. slower velocities.

## 2. Methods

### 2.1. Participants

A total of twenty-nine women and men between the ages of 18 and 30 years and twenty-three women and men between the ages of 65 and 85 years volunteered to participate in the study (see Table 1 for demographic information). A prior power analysis was conducted using G*Power 3.1.9.7 (power = 0.8, α = 0.5) and similar asymmetry studies that evaluated lower limb muscle strength and power [1,18]. We found that a sample size of *n* = 22 with an effect size of 0.66 for young adults, and *n* = 24 with an effect size of 0.62 for old adults was needed to achieve the predetermined power level. Recruiting was conducted through posted flyers and word of mouth. The eligibility criteria required participants to be either 18–30 years old or 65–85 years old, apparently healthy, generally recreationally active, and have had no major lower limb injury or surgery in the last year. Also, participants could not have any neuromuscular disease (e.g., MS, ALS etc.) and could not require the use of any assisted walking devices. They were excluded if they had any of the following: BMI below 18 or above 35, regularly participate in resistance training (>3 times per month), walk less than 1 h or more than 7 h per week or run more than 5 h per week, or were pregnant or expecting to be pregnant within 2 weeks of starting the study. Finally, participants could not be a lower limb amputee and were not allowed to participate if their physician recommended against strenuous physical activity. This study was approved by the Utah State University Institutional Review Board (#13262), and all participants read and signed an informed consent document prior to any testing.

### 2.2. Research Design

**Figure 1:**
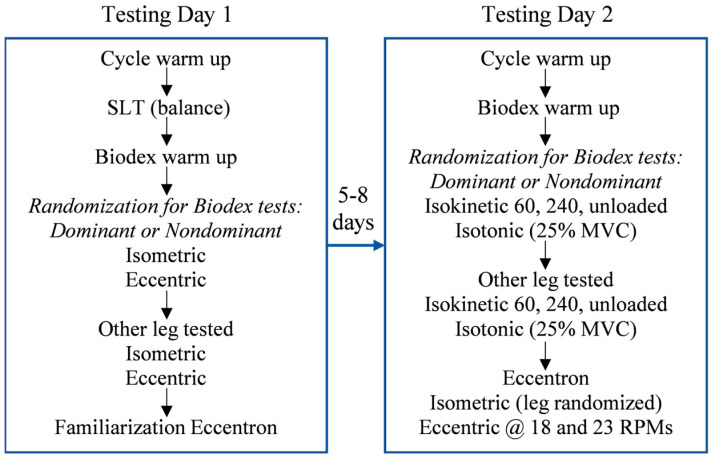
Schematic diagram showing the research design and flow of the testing. SLT = Single leg balance test. For isokinetic 60, 240, and unloaded velocities, the testing order was randomized.

The study was implemented using a cross-sectional design. Participants visited the laboratory on two separate occasions separated by 5–8 days, at the same time of day (±2 h), to limit the effects of fatigue and circadian rhythm on performance. Figure 1 shows the testing schedule.

### 2.3. Laboratory Visit I

Following the informed consent, participants completed a demographic and health history questionnaire and a modified version of the Waterloo Footedness Questionnaire to determine the dominant leg [24]. Participants then completed a physical activity questionnaire that included measures of aerobic (walking, jogging, running) and structured resistance exercise to determine weekly physical activity level. Height and body mass were then assessed using a stadiometer and calibrated scale. Before testing, participants performed a warmup consisting of cycling for 2 min on a cycle ergometer at 50 watts [25]. Lower extremity balance function (time to failure) was assessed using the SLT for which the order of testing was randomized for the dominant and non-dominant legs [15,26,27,28,29]. For this test, participants stood unsupported, with eyes closed, hands on the hips, and on one leg for as long as possible for a maximum of 120 s [27,28]. To assist in the case of balance failure, a chair was placed 1.5 feet in front of the participant and a researcher was directly behind the participant at all times. Participants were timed from when the foot left the floor to any of the following events: the foot touched the ground, the supporting foot noticeably shifted, their eyes opened, the suspended foot touched the supporting leg, a hand was removed from the hip, or if the maximum allotted time (120 s) was reached. Time to failure was recorded in seconds. Three trials per leg were performed while alternating the legs, and the representative score for each leg was the average of the three trials.

Participants were then seated on a Biodex dynamometer (Biodex System 3, Biodex Medical Systems, Shirly, NY, USA) with restraining straps placed over the waist, chest, and thigh in accordance with manufacturer guidelines. The center of the knee joint was aligned with the rotational axis of the dynamometer and the lever arm was secured to the lower leg at approximately 5 cm above the malleolus. The protocol randomized the order of the leg testing and remained on that leg through all muscle actions/velocities before being switched to the other leg for identically repeated testing. Participants then performed a muscle-specific warmup of three to five isokinetic contractions of knee extension at ~75% of perceived maximum effort at a velocity of 150 deg/s. Participants then performed three to five single-joint maximal voluntary isometric contractions (MVIC) of the knee extensors at 60 degrees of knee flexion, with a one-minute rest allotted between each trial [30]. Three to five maximal single-joint eccentric contractions were then performed at slow (60 deg/s) and relatively fast (120 deg/s) velocities [2,31].

Participants then performed an unrecorded familiarization protocol of approximately 12–18 repetitions for each leg of multi-joint eccentric muscle actions on the Eccentron at 18 RPMs.

### 2.4. Laboratory Visit II

Following a period of 5–8 days, participants reported back to the laboratory, where they began the session by performing the same general warmup as was carried out on visit 1. Participants then performed the same muscle-specific Biodex warmup as was carried out on visit 1. For the dynamometer testing protocol on visit 2, participants performed sets of five single-joint maximal concentric isokinetic muscle actions at 60 deg/s, 240 deg/s, and at an unloaded and unconstrained velocity (500 deg/s) on the Biodex [12,14]. The velocity order was randomized. Participants then performed three trials of isotonic single-joint muscle actions at 25% of their MVIC, which was determined from the first visit [32]. The leg order was randomized at the beginning of the Biodex testing and remained on that leg through all muscle actions/velocities before being switched to the other leg for identically repeated testing. Following 3–5 min of rest, participants were seated on a multi-joint dynamometer (Eccentron, BTE Technologies Inc., Hanover, MD, USA). The leg order of testing was randomized for the MVICs for the multi-joint testing. Participants performed three to five maximum multi-joint MVICs at 60 degrees of knee flexion, alternating legs, with a 1-minute rest period provided between trials.

Following a 2-minute rest period, the participants performed multi-joint isokinetic eccentric maximal voluntary contractions (MVCs) on the dynamometer at two velocities, which were randomly administered. The multi-joint eccentric test used the standard Eccentron test protocol which involved the machine providing 12 alternating cycles (6 cycles per leg) in which the pedal moved toward the participant, and they were instructed to resist the pedal movement by pushing into it as hard as possible. Two tests were conducted (2 min apart), one at 14 RPMs, which is a relatively slow velocity on this machine, and another at 23 RPMs, which is a moderately fast velocity [33].

### 2.5. Data Analysis

A Biopac data acquisition system (MP150, Biopac Systems Inc., Santa Barbara, CA, USA) was used to collect the torque, force, and velocity signals from the Biodex and Eccentron (force only) at 2000 Hz. Custom written software (LabVIEW 2021, National Instruments, Austin, TX, USA) was used to process the data in accordance with previous methods (Spencer et al., 2023) [25]. Briefly, the raw signal (V) was scaled to units (Nm and deg/s for the Biodex, and N for the Eccentron signals) and filtered using a zero phase, fourth-order Butterworth filter with a 50 Hz low-pass cut-off frequency [34]. The isometric peak torque or peak force (BD-PT-ISM) for Biodex and EC-PF-ISM (for Eccentron) was calculated as the highest 500 ms epoch across the torque– or force–time curve. RTD or RFD was calculated as the linear slope at 200 ms (BD-RTD200-ISM or EC-RFD200-ISM for the Eccentron) from onset, which was determined as 7.5 Nm (Biodex) [11] or 10 N (Eccentron) above baseline. For the isokinetic and isotonic torque signals, gravity-correction was performed to account for limb mass in accordance with the procedures previously described for the dynamic Biodex variables [35]. Isokinetic concentric and eccentric PT at 60 (slow) and 240 (fast) deg/s (BD-PT-CON60, BD-PT-CON240) and 60 (slow) and 120 (fast) deg/s (BD-PT-ECC60, BD-PT-ECC120), respectively, were calculated as the mean value of the highest 25 ms epoch between the 60 and 30 degree range of the torque–time curve for the Biodex variables. The isotonic PT (BD-PT-IST) was also calculated in this manner and the peak velocity attained during the isotonic movements was also quantified (BD-PV-IST). The peak unloaded velocity (BD-PV-UNL) was determined as the highest velocity attained during the unloaded concentric contractions similar to our previous study [14]. Peak power (PP) was calculated from the LabVIEW computed power (i.e., derived by multiplying the torque and velocity signals) signal. The slow and fast concentric PP (BD-PP-CON60, BD-PP-CON240) as well as the isotonic PP (BD-PP-IST) was calculated as the highest 25 ms epoch across the isokinetic or isotonic power-time curve. For the Eccentron slow and fast isokinetic eccentric muscle actions, the eccentric PF (EC-PF-ECC14, EC-PF-ECC23) was determined as the highest 25 ms epoch across the force–time curve.

### 2.6. Statistical Analysis

Independent *t*-tests and Chi Squared tests were performed to compare the demographics between the young and old groups. Mixed factorial analyses of variance (ANOVAs) were performed for each dependent variable for limb (dominant vs. non-dominant) and age group (young vs. old) factors. When appropriate, follow-up analyses included Bonferroni-adjusted post hoc comparisons (*t*-tests). An alpha level of *p* < 0.05 was used to determine statistical significance.

## 3. Results

For the demographics, the only variable that was significantly different between the old and young groups was age (*p* < 0.001); with height, body mass, BMI, and physical activity variables not being different between groups (*p* = 0.173–0.897). Also, the Chi Squared tests showed no age group difference for gender (*p* = 0.815) or limb dominance (*p* = 0.387).

Due to a few isolated issues for some MVCs, the following variables represented less than the full sample: BD-PV-UNL (young *n* = 28), BD-PT-ECC120 (old *n* = 22), SLT (old *n* = 22), BD-RTD200-ISM (old *n* = 22), BD-PT-CON240 (old *n* = 22), and BD-PP-CON240 (old *n* = 22).

All the muscle function variables in the study are presented in Table 2.

### 3.1. Peak Torque and Peak Force

For BD-PT-ECC, there were no significant interactions for the 60 (*p* = 0.774) or 120 (*p* = 0.861) deg/s velocities, respectively. There were also no main effects for the limbs (*p* = 0.530 and 0.506). For BD-PT-CON240, there was no interaction (*p* = 0.297) or main effect (*p* = 0.770). For BD-PT-CON60, there was technically no interaction, although it should be noted that *p* was very close to significance (0.051). However, follow-up pairwise comparisons for BD-PT-CON60 revealed there were no significant limb differences for either the young (*p* = 0.161) or old group (*p* = 0.134). For BD-PT-CON60, there was no significant main effect (*p* = 0.848). For BD-PT-ISM and BD-PT-IST, there were no interactions (*p* = 0.559 and *p* = 0.088, respectively). There were also no main effects for either of these variables (*p* = 0.912 and 0.936).

For EC-PF-ECC, there were no interactions for the 14 (*p* = 0.757) or 23 (*p* = 0.397) RPM velocities, respectively. There were also no main effects (*p* = 0.214 and 0.829). There was also no significant interaction (*p* = 0.406) or main effect (*p* = 0.330) for the EC-PF-ISM variable.

### 3.2. Power

**Figure 2:**
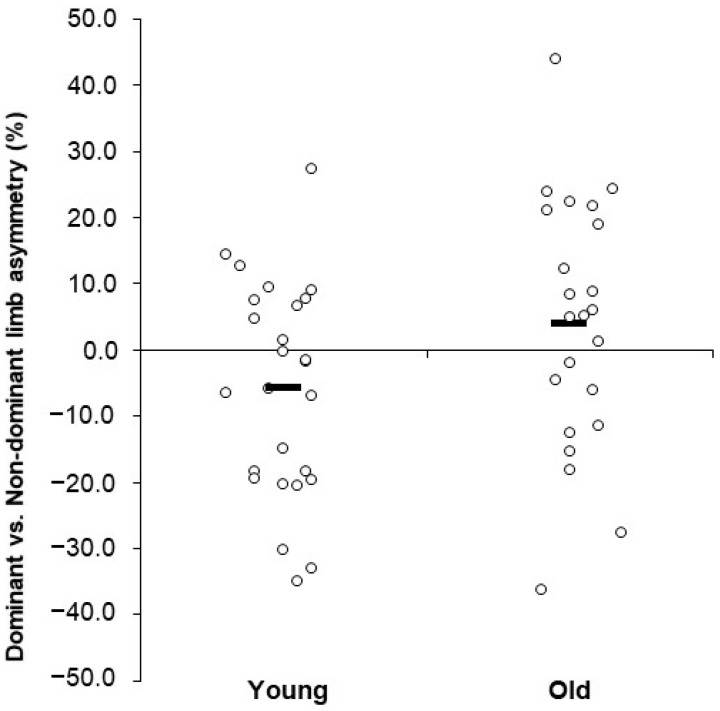
Scatterplot showing the individual limb differences (asymmetry %) for the young and old participants for the Biodex concentric peak power at 60 deg/s variable. Note: This variable showed a significant group × limb interaction, with the young showing greater values for the non-dominant vs. the dominant limb, whereas there was no limb difference for the old group. Horizontal bars are group means.

For BD-PP-CON60, there was a significant group × limb interaction (*p* = 0.010). Follow-up analyses revealed that the young group had greater PP for the non-dominant limb than for the dominant limb (*p* < 0.001), but there were no limb differences for the old group (*p* = 0.965; Figure 2 shows a scatterplot of this data). For BD-PP-CON240, there was no significant interaction (*p* = 0.070) or main effect (*p* = 0.064). For BD-PP-IST, there was no significant interaction (*p* = 0.700) or main effect (*p* = 0.129).

### 3.3. RTD and RFD

For BD-RTD200-ISM, there was no significant interaction (*p* = 0.888) or main effect (*p* = 0.664). For EC-RFD200-ISM, there was no significant interaction (*p* = 0.521) or main effect (*p* = 0.370).

### 3.4. Peak Velocity

For BD-PV-IST, there was no significant interaction (*p* = 0.591), but there was a significant main effect for the limbs (*p* = 0.046). The follow-up analysis revealed that the dominant limb was higher than the non-dominant limb, collapsed across the age groups (*p* = 0.046). For BD-PV-UNL, there was no significant interaction (*p* = 0.330) or main effect (*p* = 0.707).

### 3.5. Single-Leg Stance Time

For SLT, there was no significant interaction (*p* = 0.288) or main effect (*p* = 0.238).

## 4. Discussion

The primary results of this investigation indicated the following: (1) muscle function asymmetries of the leg extensor muscles were largely not significantly present across a multitude of modes, velocities, and variables, for either young or old populations; (2) a statistically significant asymmetry was present specifically only for the young, but not the old group, in concentric peak power at a slow velocity; and (3) the only measure that demonstrated a statistically significant asymmetry for both groups was the isotonic peak velocity variable. Based on these results, we must reject our hypothesis as the asymmetries of rapid force variables were not more prevalent for older adults than their youth counterparts.

For the majority (15 of 17) of the presently assessed variables (across modes and velocities), there were no statistically or functionally (≥10%) significant limb differences for either age group. These findings are similar to previous research that found little to no power, strength, or balance functional asymmetries in the quadriceps for young, healthy adults [18,19,21,36,37,38]. However, it should be noted that some of these studies found statistically significant asymmetries in isometric peak torque [18,19]. Previous research has also found statistically, but not functionally, significant differences in isometric strength and balance asymmetries in old healthy adults [19,39]. Note: Table 3 displays comparative asymmetry outcomes of the current study versus the core studies from the related asymmetry literature.

Interestingly, a considerable amount of previous research has found statistically significant asymmetries in isometric strength in young adults, as well as asymmetries in power and isometric and isokinetic strength during various modes and velocities for old adults [1,2,5,6,18,19,20,22,23]. While many of the studies also found functional asymmetries, some did not for measures of isokinetic strength at various velocities in the young and power in the old adults [1,18].

The reasons for these discrepancies, although unknown, could be due to differences in measurement type, sample size, or participant characteristics. For example, studies that found power and 1 RM strength asymmetries greater than 10% utilized the Nottingham Power Rig or pneumatic strength training equipment [1,2,6,20,22], which are different in form, function, and movement pattern than the types of isokinetic dynamometer assessments in the present study. Additionally, a previous key study that found significant power asymmetries in healthy old adults utilized a relatively small sample of 15 subjects (Skelton, 2002) [1], compared to that of the present study (*n* ≥ 22 subjects). Upon further inspection of the study’s results, although the data reached statistical significance (*p* < 0.05), the number of subjects with asymmetries > 10% made up only a small portion of the already small sample (2 of 15 subjects). Further, the current study may have yielded different results in asymmetries compared to these prior studies for old adults as a result of the recreationally active and relatively healthy older adult population (BMI 18–35) that was examined. Based on previous research, increased physical activity may be associated with delayed functional impairment, prevention of muscular imbalances of knee extensors, and greater physical health [17,19,40]. Further, unlike the upper limbs, the lower limbs are generally used in a manner of bilateral symmetry during daily activities (e.g., walking, standing, etc.), which likely limits the effects of dominance-related differences. For example, unlike with upper limb dominant tasks that mostly involve the dominant limb being active while the non-dominant limb remains relatively inactive, with the lower limbs, when the dominant limb is performing a specific action, the non-dominant limb commonly remains active in providing stabilizing and load-bearing support [38]. This may further explain why the current study found no significant difference in lower limb balance function (SLT) for either age group.

A unique finding of this investigation was that there was a statistically significant and nearly significant group × limb interaction for the concentric slow (60 deg/s) variables (e.g., BD-PP was *p* = 0.010 and BD-PT was *p* = 0.051; see Figure 2). The significant interaction was a result of a statistically and functionally significant difference between peak power produced in the dominant and non-dominant limb in the young group, where the non-dominant limb showed 12.2% greater PP values compared to the dominant limb, whereas the old group had a non-significant 0.19% greater PP in their dominant limb. These findings are consistent with previous research that found asymmetry patterns do not always follow limb dominance patterns [1]. However, these findings are contrary to previous research that found greater asymmetries in the old group compared to the young group [2,22]. This is a rather surprising finding and the reason for it is unknown. Although speculative, it is possible that asymmetries were present in the young group due to unequal mechanical stimuli as a result of preferential use of one limb over another [23]. Such is a plausible scenario where perhaps the young individuals may be using the non-dominant limb to a greater extent than older persons, such as for certain support tasks associated with sport, recreation, or exercise-specific activities. Further, although a speculative hypothesis, as lower limb asymmetries may be beneficial for these activities as a consequence of sport-specific adaptation responses, the older group may not have expressed asymmetries possibly due to a history of inactivity in these activities, which may have led to detraining of the limbs, and thereby could have attenuated the presence of activity-specific asymmetries. More research is needed examining this theory, specifically in regard to how differently aged populations may use their non-dominant limbs in different ways across daily living activities and athletic-related tasks.

Another key finding from the present study was that there was a statistically significant age-related asymmetry for the isotonic peak velocity variable. Although functional asymmetries were not met (5.1% for old, 2.1% for young) for isotonic peak velocity, it is noteworthy that this is the only variable to show a higher dominant limb asymmetry for both age groups, and thus the only one that revealed a statistically significant asymmetry, specifically for the old group. This is interesting as contraction velocity is not commonly assessed. This is further a novel finding as, to our knowledge, no previous study has examined contraction velocity variables with regards to limb dominance. Notably, isotonic tests are considered more functionally relevant in older adults as compared to isometric or isokinetic tests [32], perhaps making them more sensitive to subtle changes in performance and thereby leading to a heightened capacity to provide a holistic assessment of muscle function. This may explain why age-related effects were revealed during the isotonic peak velocity mode and not detected in isometric or most isokinetic modes. Based on this finding, isotonic tests may be a rather sensitive measure for muscle function, and future research may consider incorporating this type of testing into dynamometer-based muscle function test batteries in order to determine whether it may have the potential to be a useful measure when assessing subtle performance differences in clinical populations.

The present study has some limitations that are noteworthy. First, this study’s participants represent recreationally active adults, so these results may not apply to more sedentary, clinical, or mobility-limited populations. Further, unlike its concentric counterpart, this study was unable to utilize a very fast eccentric velocity on account of safety concerns. As such, the eccentric fast modes on the Biodex may not be functionally matched to their concentric counterparts. Further, unlike the Biodex, the Eccentron is unable to assess concentric measures. Consequently, unlike the eccentric modes, this study had no way to match concentric modes from the Biodex on the Eccentron. Further, although the single-leg stance test is a commonly utilized balance measure, it is a relatively simple measure of balance. As such, asymmetries may have been better detected if a more complex balance test (such as center of pressure detection) was utilized. Therefore, future studies may benefit from using matched modes and mode velocities and more complex balance tests to better capture asymmetries in these groups. Future work may also consider the complex interactions involving muscle asymmetries relating to the longitudinal age-related declines in force and power measures as well as how upper and lower body structural changes, such as disproportionate upper and lower body mass and/or muscle mass changes, may impact inter-limb imbalances.

## 5. Conclusions

In conclusion, this study yielded results that may help focus efforts of determining asymmetries in young and older adults. The findings of this investigation indicate that there is largely no age-related asymmetry of the lower limbs across a range of variables and modes. However, this study found significant asymmetries in concentric peak power produced at a slow velocity for young adults that were not present in old adults. Moreover, there were statistically significant asymmetries for isotonic peak velocity for both age groups, which perhaps showed the sensitivity of this uncommon measure in detecting minimally present muscle function imbalances. Further, this study is likely the first of its kind to assess and detect velocity-specific asymmetries in an aging context. These findings may help inform future studies that aim to assess lower limb asymmetries in regards to what types of measurements and modes may be best suited for the most effective testing.

## Figures and Tables

**Table 1:** Demographic data of the young and old groups. Data are means (SD) or percent.

Variable	Young (*n* = 29)	Old (*n* = 23)
Age (yrs) *	21.45 (3.02)	77.00 (4.60)
Height (cm)	173.16 (10.86)	168.91 (11.16)
Body Mass (kg)	72.22 (16.91)	72.91 (21.68)
BMI (kg/m^2^)	23.88 (3.91)	25.15 (4.91)
Female (%)	62.07	60.87
Physical Activity (h/wk)	2.03 (2.36)	2.13 (2.44)
Right Limb Dominant (%)	93.10	82.61

* Indicates significantly different between groups at *p* < 0.05.

**Table 2:** Mean (SD) for all variables across modes and velocities for the dominant and non-dominant limbs in the young and old groups.

		Young		Old	
Variable	Mode/Velocity	Dominant	Non-Dominant	Dominant	Non-Dominant
BD-PT	ISM	170.63 (62.89)	172.61 (57.64)	123.10 (49.46)	120.20 (54.46)
BD-PT	IST	64.20 (16.12)	66.57 (13.74)	63.57 (27.54)	60.97 (29.82)
BD-PT	ECC60	212.82 (63.07)	219.08 (67.39)	164.43 (54.13)	166.77 (68.81)
BD-PT	ECC120	211.03 (64.42)	216.27 (66.04)	164.23 (56.74)	167.28 (61.60)
BD-PT ^†^	CON60	140.76 (45.47)	146.84 (52.75)	98.77 (41.90)	93.76 (40.59)
BD-PT	CON240	82.50 (36.66)	84.19 (36.99)	59.63 (24.03)	56.63 (24.56)
EC-PF	ISM	1680.42 (535.30)	1675.95 (551.47)	1083.15 (384.24)	1027.35 (376.63)
EC-PF	ECC14	1901.71 (564.86)	1866.06 (512.04)	1217.76 (457.80)	1158.70 (418.38)
EC-PF	ECC23	1868.41 (551.04)	1895.13 (536.33)	1161.19 (435.66)	1145.28 (455.46)
BD-PP	IST	454.74 (181.18)	444.00 (158.68)	252.79 (120.48)	234.85 (105.31)
BD-PP ^ⱡ^	CON60	140.31 (46.99)	159.82 (55.83) **	103.03 (45.84)	103.23 (43.48)
BD-PP	CON240	321.92 (147.18)	359.97 (157.01)	240.63 (106.18)	241.12 (102.47)
BD-RTD200	ISM	537.52 (247.97)	550.01 (212.29)	380.51 (186.14)	386.87 (201.63)
EC-RFD200	ISM	1688.73 (979.65)	1773.30 (789.28)	1271.80 (632.40)	1285.84 (549.87)
BD-PV *	IST	388.49 (59.62)	380.37 (56.80)	272.86 (50.04)	258.89 (37.65)
BD-PV	UNL	439.12 (60.91)	447.34 (51.15)	344.77 (60.19)	341.12 (51.71)
SLT	N/A	15.89 (16.33)	19.41 (18.93)	2.16 (0.98)	2.35 (1.64)

Note: BD = Biodex; EC = Eccentron; ECC = eccentric; CON = concentric; ISM = isometric; IST = isotonic; PT = peak torque; PF = peak force; PP = peak power; RTD = rate of torque development; RFD = rate of force development; PV = peak velocity; UNL = unloaded concentric velocity; SLT = single-leg stance time. * indicates a significant effect for limb, collapsed across age group. ^ⱡ^ indicates a significant group × limb interaction (see text for explanation). ^†^ indicates *p* = 0.051 for the group × limb interaction. ** indicates higher vs. dominant limb for the young group only. *p* < 0.05 for determining statistical significance.

**Table 3:** Comparative table illustrating the findings on muscle function asymmetries between the current study and core asymmetry studies from the literature.

Study	Young Adults			Older Adults	
	No Asymmetry: Balance, Power, Isometric and/or Isokinetic Strength	Asymmetry: Balance, Power, Isometric and/or Isokinetic Strength	No Asymmetry: in Balance, Power, Isometric and/or Isokinetic Strength	Asymmetry in Balance, Power, Isometric and/or Isokinetic Strength	Older with More Asymmetry than Young
Current Study	X	X	X	X	
[2]	-	-	-	X	X
[1]	-	-	-	X	
[5]	-	-	-	X	
[17]	-	-	-	X	
[19]		X		X	
[6]	-	-	-	X	
[18]	X	X			
[20]	-	-	-	X	
[21]	X				
[22]	-	-	-	X	X
[23]	-	-	-	X	
[36]	X				
[37]	X				
[38]	X				
[39]	-	-	-	X	

## Data Availability

The raw data supporting the conclusion of this article will be made available by the authors upon request.
